# Osteoclastogenesis of human peripheral blood, bone marrow, and cord blood monocytes

**DOI:** 10.1038/s41598-023-30701-0

**Published:** 2023-03-07

**Authors:** Ella Vuoti, Petri Lehenkari, Juha Tuukkanen, Virpi Glumoff, Elina Kylmäoja

**Affiliations:** 1 Institute of Cancer Research and Translational Medicine, Department of Anatomy and Cell Biology, Medical Research Center, University of Oulu, P.O. Box 5000, 90014 Oulu, Finland; 2 Research Unit of Biomedicine, University of Oulu, Oulu, Finland

**Keywords:** Osteoclasts, Haematopoietic system, Preclinical research, Stem-cell research, Haematopoietic stem cells, Stem-cell differentiation, Bone

## Abstract

Osteoclasts are multinucleated bone resorbing cells that can be differentiated from human monocytes in vitro. There are few studies comparing osteoclastogenesis of different monocyte sources. We compared monocytes from human bone marrow (BM), peripheral blood (PB), and umbilical cord blood (CB) and their osteoclastogenic potential by culturing them with RANKL (20 and 80 ng/ml) and M-CSF (10 ng/ml) for 14 days. We also cultured cells without growth factors, as umbilical cord blood monocytes have been reported to be able to fuse spontaneously into osteoclasts. The data was analysed on d4, d8, d11, and d14. After culture with RANKL and M-CSF, all types of cell cultures developed TRACP -positive multinuclear cells that were able to form resorption pits on human bone slices. Only occasional multinuclear cells and small infrequent resorbed areas could be found in PB and CB-derived cultures without growth factors. BM-derived cells formed greater resorption areas than PB- and CB-derived monocytes. The greatest monocyte population in BM samples were intermediate (CD14^++^CD16^+^) and in PB and CB classical monocytes (76.3% and 54.4%, respectively). In conclusion, our data demonstrates that bone resorbing osteoclasts can be differentiated from BM, PB and CB. However, the osteoclast precursor origin can affect the osteoclast properties and function.

## Introduction

It is well documented that monocyte/macrophage lineage osteoclast precursors can be isolated from bone marrow (BM) or peripheral blood (PB) and differentiated into multinuclear bone resorbing osteoclasts in vitro^1–4^. In addition, several studies have shown that similar precursors are present in umbilical cord blood (CB), and that they are also capable of fusion into osteoclasts in vitro. Differentiation has been documented after stimulation with the commonly used osteoclastogenic factors such as macrophage colony-stimulating factor (M-CSF), receptor activator of nuclear factor kappa-B ligand (RANKL), and 1,25-dihydroxyvitamin D3 or in a coculture with osteoblast lineage cells supposedly producing these factors^5–13^. However, there are few studies comparing the differences of the generated multinuclear cells or osteoclasts from the different sources. Two studies comparing multinuclear giant cell formation from adult PB and CB monocytes reported that CB monocytes did not differentiate into multinuclear cells as efficiently as PB monocytes, presumably depicting the immature pool of monocytes present in CB^14,15^. In another study, osteoclasts were formed in PB and CB monocyte cultures after stimulation with RANKL and M-CSF^5^. Osteoclast numbers were not compared in this study, but the authors reported that interleukin-33 (IL-33) induced osteoclastogenesis only in adult PB monocyte cultures but not in CB cultures, indicating that there are differences in how the different monocytes respond to growth factors or cytokines. When effects of estrogen on osteoclastogenesis from BM and CB monocytes were studied, it was noted that estrogen inhibited osteoclastogenesis in both cell types^10^. In this study, the number of TRACP-positive multinuclear cells was slightly higher in BM cultures, but the expression of osteoclast-associated antigen vitronectin receptor was higher in CB cultures. Based on the higher expression of macrophage cell surface antigens CD11b and CD14 in CB monocytes the authors suggested that CB contains a higher number of osteoclast precursor cells.

The aforementioned studies suggest that the CB monocyte pool does not fuse into multinuclear cells as efficiently as PB or BM monocytes. However, the osteoclasts from CB monocytes have been reported to be more resistant to apoptosis compared to osteoclasts from PB monocytes^12^. In this study, no differences were observed in the resorption activity or expression of osteoclast markers in osteoclasts generated from the two cell sources. Interestingly, in this study, the CB derived osteoclasts developed spontaneously without any osteoclastogenic growth factors. Similar results have been reported also in other studies^16,17^. The spontaneous fusion is likely the result of the active mesenchymal stromal cell population that is present in CB producing osteoclastogenic factors. The spontaneous fusion has also been shown to occur in minute amount in adult PB monocyte cultures^15^.

To our knowledge, our present study is the first study comparing osteoclastogenesis from adult PB, BM, and CB monocytes. In addition, we have studied the effects of increasing RANKL concentrations on osteoclastogenesis from these sources.

## Materials and methods

### Osteoclastogenesis from human BM monocytes

The isolation and culture protocol were modified from^18^. BM samples were obtained from hip replacement surgery patients in Oulu University Hospital. The samples were taken from the femoral bone marrow after removing the femoral neck and the sample was transferred into a tube containing culture media. No anticoagulant was used. Patients were 43–75-year-old men and women who gave a written informed consent. The number of BM donors was 8. The patient samples used for different experiments are listed in Table 1. The study was approved by the Ethical Committee of The Northern Ostrobothnia Hospital District and all experiments were performed in accordance with the Declaration of Helsinki. BM sample was precultured in α-MEM (Corning Life Sciences, Tewksbury, MA) containing 10% FBS (Biowest, Riverside, MO), 100 IU/ml penicillin and 100 µg/ml streptomycin and 24 mM Hepes buffer (Sigma-Aldrich, St. Louis, MO) at + 37 °C (5% CO_2_, 95% air) for 1–2 days. After this, medium containing the non-adherent cells was collected, diluted 1:1 in PBS and layered over (1:1) Ficoll-Paque Premium solution (GE Healthcare, Little Chalfont, UK). The samples were centrifuged at 400×*g* for 35 min following the manufacturer’s protocol. Mononuclear cell layer was collected and centrifuged twice at 190×*g* for 10 min in PBS, and finally suspended in α-MEM (Sigma-Aldrich). 300,000 cells (9.4 × 10^5^ cells/cm^2^) were layered on sonicated human cortical bone slices (0.28 cm^2^) in 96-well plates (Cellstar; Greiner Bio-One, Kremsmünster, Austria). The cell seeding density was optimized for osteoclastogenesis from our cell sources. The slices were cut from anonymous bone samples acquired from clinical bone bank held in Oulu University Hospital, city of Oulu, Finland. Special National Supervisory Authority for Welfare and Health (Valvira) granted a permission for use of aged cadaver specimens for research purposes, decision 8.5.2009, diary number 2240/05.01.00.06/2009. Cells were cultured in α-MEM (Corning Life Sciences) containing 10% FBS, 100 IU/ml penicillin and 100 µg/ml streptomycin. Osteoclastogenesis was induced with 20 ng/ml or 80 ng/ml RANKL (PeproTech EC, UK) and 10 ng/ml M-CSF (R&D Systems, Minneapolis, MN). Cell culture media were collected on days 4, 8, 11 and 14 and stored at − 80 °C. Half of the media was changed on days 4 and 11 (100 µl/well). On day 8, all media was changed (200 μl/well). Cells were cultured at + 37 °C (5% CO_2_, 95% air) for 14 days.

**Table 1:** Patient samples used in each experiment.

Donor	Sex	Age	Cell and nuclei numbers, resorption	FACS	Cytokine analysis
1	M	72*	BM		BM
2	M	44	BM		BM
3	M	69	BM		BM
4	F	70	BM + PB	PB	BM + PB
5	M	43	PB		PB
6	F	67	PB	BM + PB	PB
7	M	73	PB	BM + PB	PB
8	M	75		BM + PB	
9	M	68		BM + PB	
1	F	37 + 0**	CB		CB
2	F	39 + 1	CB		CB
3	F	39 + 1	CB		CB
4	F	39 + 4	CB		CB
5	F	38 + 5		CB	
6	F	37 + 2		CB	
7	F	39 + 1		CB	

*Years.

**Gestational age at birth; weeks + days.

### Osteoclastogenesis from human PB monocytes

PB monocytes were collected from whole blood sample from the hip replacement surgery patients shortly before the operation. The patients gave a written informed consent before donating the samples. The number of PB donors was 6. Lithium heparin vacuum tubes were used to collect the blood. Ficoll-Paque Premium gradient centrifugation was performed for the diluted (1:1; PBS) blood sample to isolate mononuclear cells. PB monocytes were cultured and differentiated in the same manner as the BM monocytes as described earlier.

### Osteoclastogenesis from human CB monocytes

CB monocytes were collected from umbilical cord blood vessels after full term elective caesarean sections. The mothers were healthy and gave a written informed consent before donating the samples. The number of mothers participating in the study was 7. Blood was collected in tubes containing heparin 100 IU/ml (Leo Pharma, Vantaa, Finland) 1:10 ratio to collected blood amount. Ficoll-Paque Premium gradient centrifugation was performed for the diluted (1:1; PBS) CB sample as described earlier. Cell culture and differentiation were done in the same manner as the BM and PB samples.

### Counting of multinuclear cells and the number of nuclei

After culturing the cells were fixed with 4% PFA in PBS for 10 min at RT. The actin cytoskeleton was stained with Alexa 488-conjugated phalloidin (200 U/ml stock diluted 1:100 in PBS; Invitrogen Europe, Paisley, UK) for 20 min at + 37 °C. Nuclei were stained with Hoechst 33258 (1 mg/ml stock diluted 1:800 in PBS; Sigma-Aldrich) for 10 min at room temperature (RT). Staining for osteoclast-specific enzyme TRACP was carried out with a commercial acid phosphatase leukocyte kit (Sigma-Aldrich) for 20 min at + 37 °C. The samples were mounted in 70% glycerol-PBS and viewed in a Zeiss Axio Scope.A1 fluorescence microscope (Oberkochen, Germany) and EC Plan Neofluar 20 × objective. Multinuclear cells with three or more nuclei were counted from each bone slice from five randomly chosen microscope fields, bone slice *n* = 4–6. The number of nuclei per cell were counted from 5 multinuclear cells from 5 randomly chosen areas. Images were taken with Nikon Eclipse E600 fluorescence microscope using Plan 20 ×/0.5 objective (Tokyo, Japan), QImaging MicroPublisher 5.0 RTV camera and QCapture 2.90.1 software (QImaging, Surrey, Canada). Confocal images were taken with Leica TCS SP8 confocal with a DMI8 microscope using LAS X 3.5.2 acquisition software. The objective used was an HC PL APO CS2 20 ×/0.75 DRY. Samples were imaged with 488 nm and 405 nm solid-state lasers; the pinhole was set to Airy 1 and scan speed to 600 Hz.

### Field emission scanning electron microscopy and measurement of resorption pit areas

Resorption pit areas were measured from Field Emission Scanning Electron Microscopy (FESEM) images with Merz grid analysis. After multinuclear cell counting, cells were detached from the slices by brushing, samples were dehydrated in ascending ethanol series and dried with a critical point drying equipment K850 (Quorum technologies, UK). Samples were coated with 5 nm platinum by Q150T ES sputter coater (Quorum Technologies) and viewed with Sigma HD VP FE-SEM (Carl Zeiss Microscopy GmbH, Germany). FESEM images were taken from three fields (voltage 5.0 kV, magnification 50 ×, area 0.039 cm^2^) from each bone slice, *n* = 3. The morphometric analysis of the pits was performed with ImageJ 1.51n software (NIH, USA) by superimposing a Merz grid with 92–96 points in semicircular lines over the image. Points in pits were counted and the percentage of resorption pits versus intact bone surface was counted. The percentage of resorbed area was normalized to multinuclear cell number (counting described above), and the average area resorbed by one cell was calculated.

### Characterisation of monocyte subsets with FACS

Ficoll-isolated monocytes (500,000 cells) from fresh PB and BM samples (after the 1–2 days preculture) were stained with 1.25 µl allophycocyanin (APC)-conjugated anti-CD14 (BD Biosciences, Franklin Lakes, NJ; Cat. No. 561383), 5 µl phycoerythrin (PE)-conjugated anti-CD16 (BD Biosciences; Cat. No. 555407), and 1.25 µl fluorescein isothiocyanate (FITC)-conjugated anti-CD3 antibodies (Invitrogen; Cat. No. 11-0036) for 20 min at RT. Ficoll-isolated CB monocytes (500,000 cells) were stained with above-mentioned amounts of APC-conjugated anti-CD14, PE-conjugated anti-CD16 and 2.5 µl FITC-conjugated anti-CD45 antibodies (BD Biosciences; Cat. No. 555482) for 20 min at RT. Samples were run on the BD Accuri 6C Plus (BD Biosciences) flow cytometer and analyzed with FlowJo v10.7.1 software (FlowJo LLC.).

### RANKL, M-CSF and OPG measurement from cell culture media

Cell culture media were collected after the experiment and stored at − 80 °C. Samples were analyzed with custom ProcartaPlex panel (Thermo Fisher Scientific, Waltham, MA) for RANKL, M-CSF and osteoprotegerin (OPG). After thawing, the media were centrifuged at 10,000×*g* for 5 min. 100 µl medium was pipetted into 96-well plates and stored overnight at + 4 °C. Samples were analyzed in duplicates following the kit instructions and using a Luminex™ MAGPIX™ Instrument system (Luminex Corporation, Austin, TX). During the analysis, plates were washed with BioTek Instruments Microplate Strip Washer EL × 50 (BioTek Instruments, Winooski, VT).

### Statistical analysis

All experiments were done with groups of *n* ≥ 4 and repeated with at least 3 independent patient samples. Statistical analyses were performed using SPSS Statistics program version 25 (IBM Corp., Armonk, NY, USA). Graphical presentations were created using OriginPro 2020b (OriginLab Corporation, Norhampton, USA). The normality of the response variables was tested with Kolmogorov–Smirnov or Shapiro–Wilk test (for experiments with *n* < 50) and histogram visualization. Statistical differences between the test groups were evaluated using Kruskal–Wallis test, and comparison between groups was done with Mann–Whitney *U* test. *p* < 0.05 was considered significant. Benjamini–Hochberg procedure was performed for comparisons of *n* > 20 *p*-values. Data are shown as means ± SEM.

## Results

### Multinuclear cells were formed in BM, PB, and CB monocyte cultures

**Figure 1:**
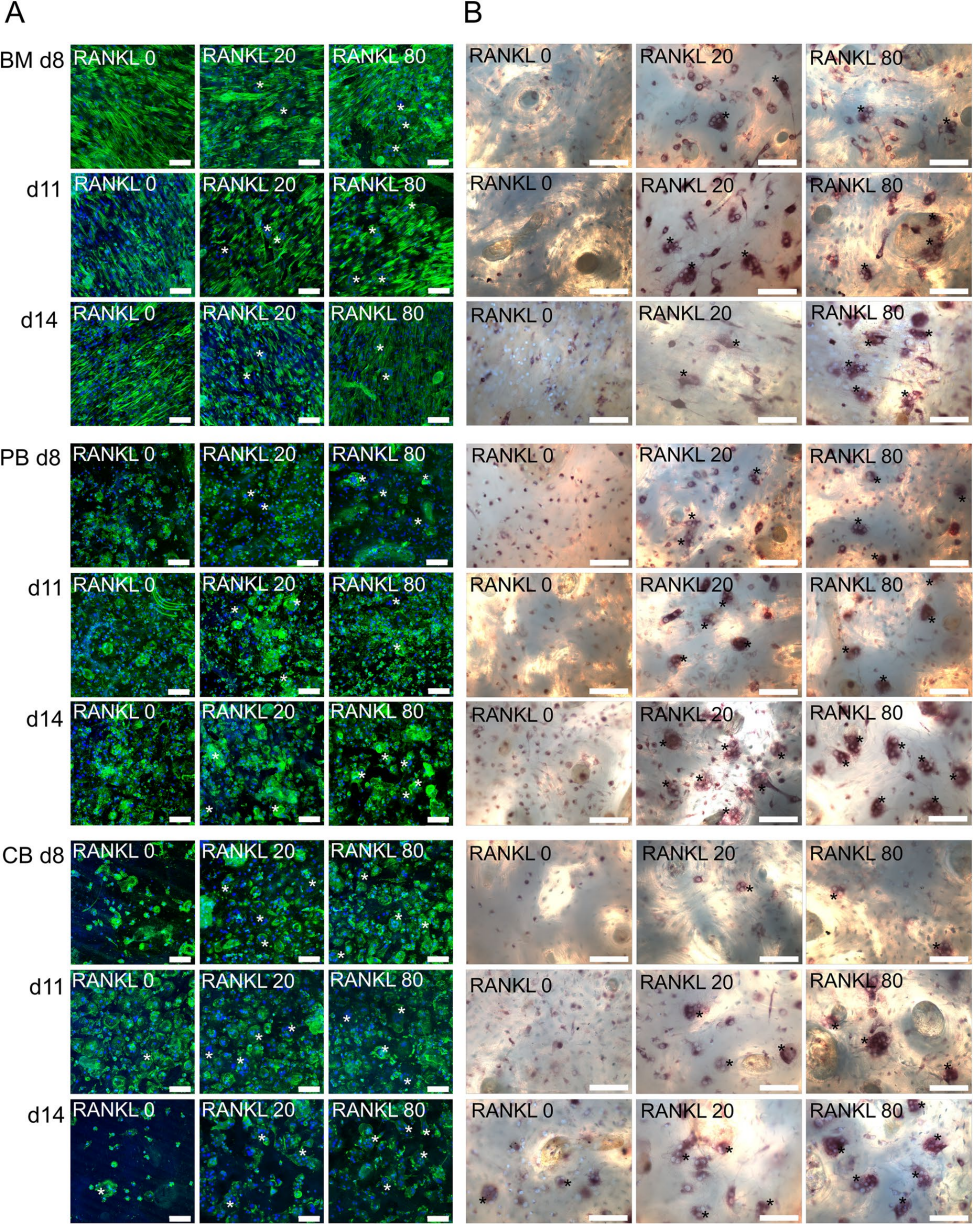
Multinuclear cell differentiation in BM, PB and CB cultures. (**A**) Confocal images of osteoclast cultures at time points d8, d11, and d14. The cells were cultured on human bone slices. The actin cytoskeleton was stained with Alexa 488-conjugated phalloidin (green) and nuclei were stained with Hoechst (blue). Images were taken with confocal microscope and 20 × objective (representative data from one patient). Multinuclear cells are marked with asterisk (*). (**B**) Combined light and fluorescence microscope images of osteoclast cultures at time points d8, d11, and d14. The cells were stained with Hoechst (blue) and acid phosphatase leukocyte kit to visualize TRACP (red). Images were taken with 20 × objective.

**Figure 2:**
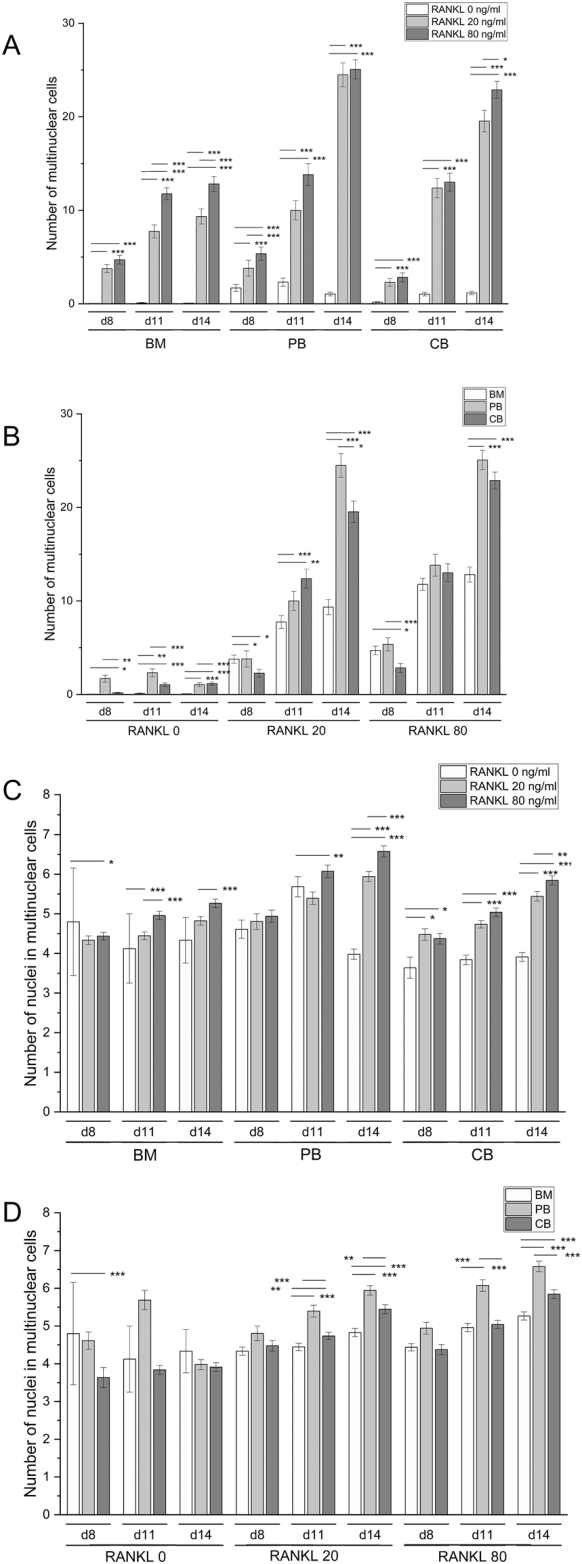
The number of multinuclear cells and nuclei in BM, PB and CB cultures. A&B. The number of multinuclear cells at different time points during the cell culture. The data in graphs A and B is same but presented in regard to cell source (**A**) or RANKL concentration (**B**) for easier comparison between the samples. (**C**) The number of nuclei in multinuclear cells. The data in graphs is pooled from 4 patients per culture type and shown as mean ± SEM. *p < 0.05, **p < 0.01, ***p < 0.001.

All types of monocyte cell cultures developed tartrate-resistant acid phosphatase (TRACP)-positive multinuclear cells after culture with RANKL and M-CSF (Fig. 1). A significant proportion of cells in BM cultures were fibroblast-shaped stromal-like cells, which were almost absent in PB and CB cultures. According to the hypothesis that cell fusion begins approximately after 1 week culture, there was a small number of multinuclear cells on day 8 in all monocyte cultures we investigated. The number of multinuclear cells increased over time in all cultures, being highest on day 14 when the cultures were fixed. Increasing RANKL concentration from 20 to 80 ng/ml had a mild effect on the number of multinuclear cells in all cell sources (Fig. 2A). At the end of the experiment, the number of multinuclear cells was significantly lower (*p* < 0.001) in BM cultures compared to PB and CB cultures (Fig. 2B). Most of the multinuclear cells in BM-derived cultures were fused by d11, whereas in PB and CB cultures cells continued to fuse between d11 and d14. Occasional multinuclear cells could be found in PB and CB-derived cultures without growth factors (i.e., spontaneous osteoclastogenesis), whereas in BM-derived cultures their numbers were very low. At the end of the experiment, the number of nuclei in multinuclear cells followed the order PB > CB > BM in RANKL-containing cultures, but the differences were minor (Fig. 2C). Increasing RANKL concentration from 20 to 80 ng/ml had a small but statistically significant (*p* < 0.001 in BM and PB, *p* < 0.01 in CB) increasing effect on the number of nuclei by d14.

### Bone marrow derived osteoclasts resorbed bone more efficiently

**Figure 3:**
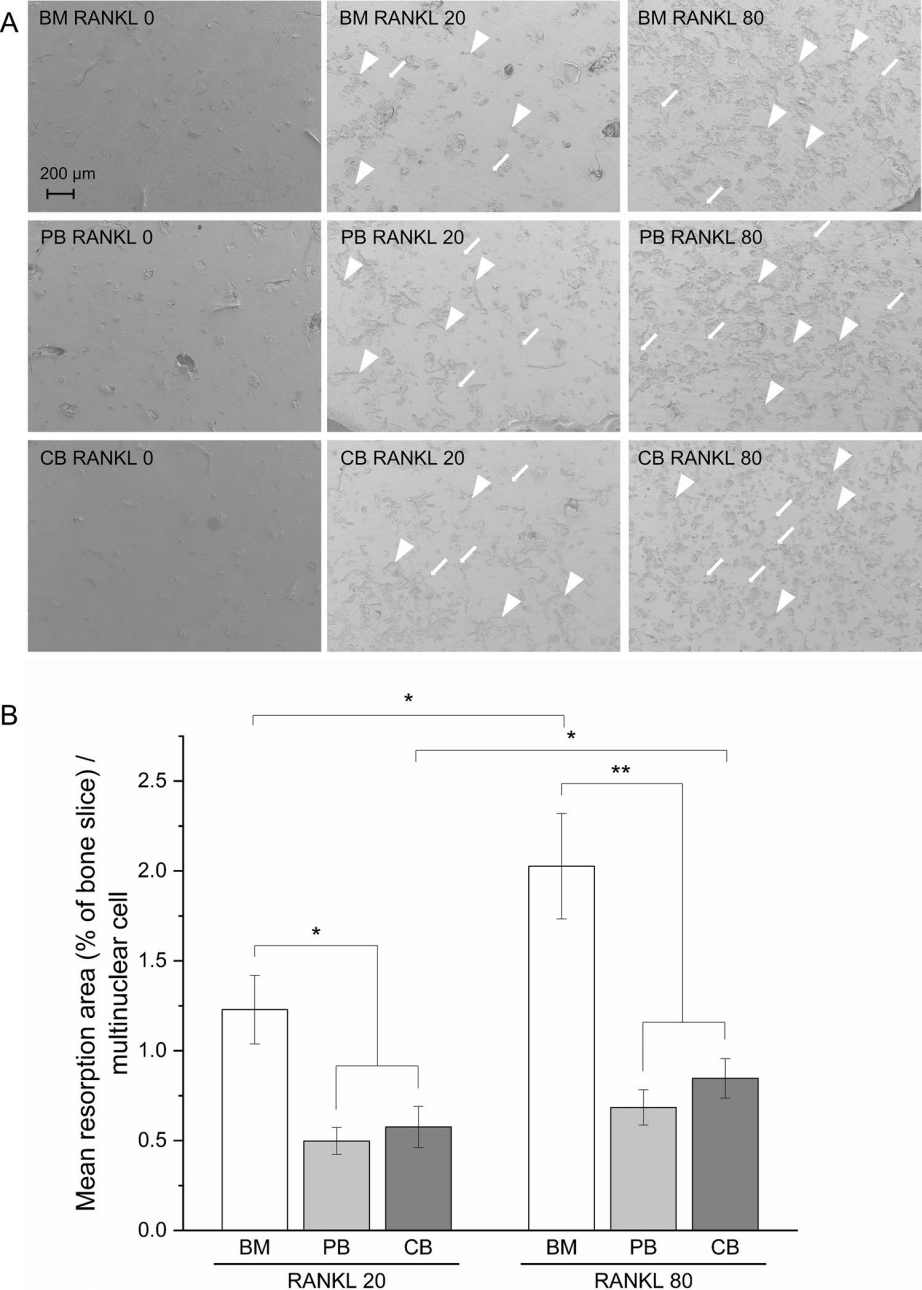
Bone resorption activity of osteoclasts differentiated from BM, PB and CB. (**A**) FESEM images of the bone slices on d14 were taken with 50 × magnification (representative data from one patient). Pit-like resorption pits are pointed with arrows and trench-like resorption pits with arrowheads. (**B**) Mean resorption pit areas measured from FESEM images on d14. The data in the graph is pooled from 4 patients per culture type and shown as mean ± SEM.

Osteoclasts from all three different sources were able to form resorption pits on human bone slices (Fig. 3A). Slices from cultures without growth factors had only few small infrequent resorbed areas, which was in line with other analyses. Increasing RANKL concentration from 20 to 80 ng/ml enhanced resorption in BM (from 1.2 to 2.0%, *p* < 0.05) and CB (0.50–0.78%, *p* < 0.05) cultures, but not PB cultures (0.50 versus 0.68%, *p* = 0.215) (Fig. 3B). The resorption area of BM-derived cells was greater than of PB- and CB-derived cells. BM-derived cells seemed to form more trench-like resorption areas, whereas resorption areas of PB- and CB-derived (0.58% in RANKL 20 ng/ml group, 0.78% in RANKL 80 ng/ml group) cells were more pit-like.

### Cytokine concentrations of the media showed a fluctuating pattern

**Figure 4:**
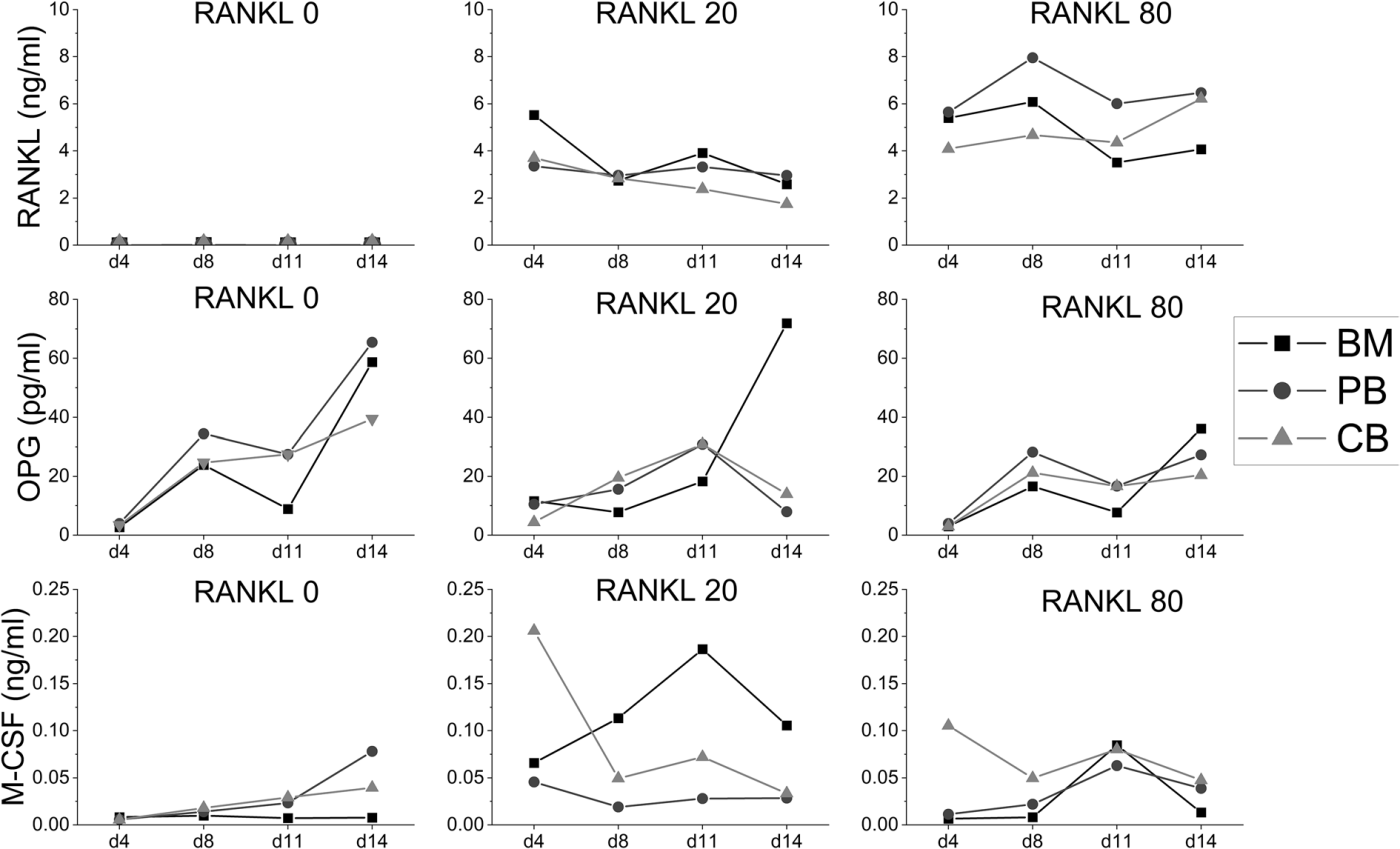
Cytokine concentrations in BM, PB and CB cultures at timepoints d4, d8, d11 and d14. RANKL numbers indicate the concentration of RANKL (ng/ml) added to the cell culture. Cell culture media were collected on the above-mentioned timepoints and analyzed with custom ProcartaPlex panel for RANKL, M-CSF and OPG. The data in the graph is pooled from 4 patients per culture type and shown as mean ± SEM.

The concentrations of RANKL, OPG and M-CSF in the culture media are shown in Fig. 4. Analysis of RANKL in culture media (upper panel) showed that the concentrations had a similar fluctuating pattern in all cell sources in RANKL 20 ng/ml and 80 ng/ml cultures. The concentrations of RANKL in cultures without added growth factors (RANKL 0 ng/ml group) were under detection limit regardless of the cell source. The results from OPG analysis (middle panel) showed that the concentrations followed similar fluctuation over time in all cell sources in RANKL 0 and RANKL 80 -cultures. In RANKL 20 group, OPG peaked (72 pg/ml) in BM cultures on d14. Concerning M-CSF in culture media (lower panel), without growth factors added (RANKL 0 ng/ml group), the concentration remained low in BM cultures but increased slightly by d14 in PB (78 pg/ml) and CB (39 pg/ml) cultures. In cultures with added growth factors (RANKL 20 & 80 ng/ml), the M-CSF concentration on d4 was significantly higher in CB (206 pg/ml and 105 pg/ml, respectively) than BM (66 pg/ml, *p* < 0.01; and 6.5 pg/ml, *p* < 0.001; respectively) and PB (46 pg/ml, *p* < 0.005; and 11 pg/ml, *p* < 0.001; respectively) cultures but declined by d8. The M-CSF-concentration elevated the most between timepoints d8 and d11 in all cell culture types when RANKL (20 or 80 ng/ml) was added to the media.

### Distribution of monocyte subsets varied between the three cell sources

**Figure 5:**
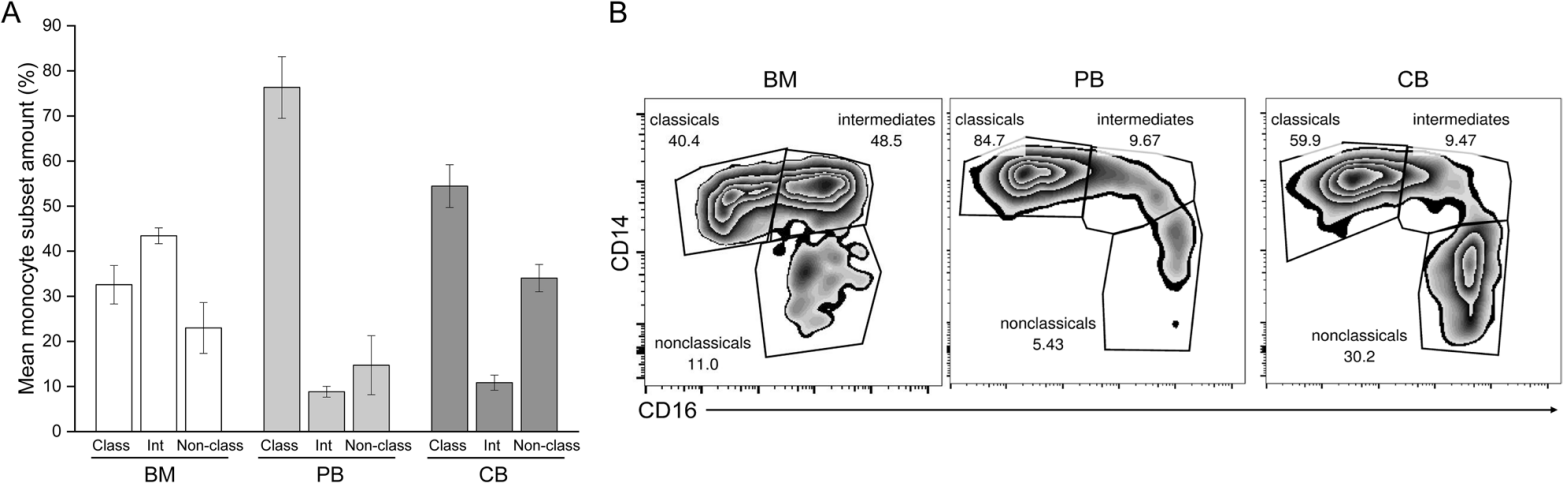
Monocyte subsets in BM, PB and CB samples before cell culture. (**A**) Proportions of monocyte subsets in the samples. Class: classical (CD14^++^ CD16^−^); Int: intermediate (CD14^++^CD16^+^); Non-class: non-classical (CD14^+^ CD16^++^) monocytes. The data in the graph is pooled from 4 patients (BM), 5 patients (PB) or 3 patients (CB) and shown as mean ± SEM. (**B**) Representative FACS-data of each sample from one patient. Ficoll-isolated monocytes from BM and PB were stained with PE-conjugated anti-CD14, APC-conjugated anti-CD16 and FITC-conjugated anti-CD3 antibodies. Ficoll-isolated CB monocytes were stained with PE-conjugated anti-CD14, APC-conjugated anti-CD16 and FITC-conjugated anti-CD45 antibodies. Samples were run on the cytometer and analyzed with FlowJo v10.7.1 software.

There was variation between BM, PB, and CB samples in the distribution of monocyte subsets (Fig. 5). The distribution of monocytes in BM was classical (CD14^++^ CD16^−^) 32.6%, intermediate (CD14^++^CD16^+^) 43.4%, and non-classical 23.0%. In PB, the distribution was: classical monocytes 74.1%, intermediate monocytes 8.9%, and non-classical monocytes 16.8%. In CB, the distribution was 54.4%, intermediate 10.9%, and nonclassical 34.0%.

## Discussion

In the present study, we compared osteoclastogenic potential of human monocytes from three different sources: BM, PB, and CB. We were able to differentiate multinuclear TRACP-positive bone resorbing cells from all three cell sources. To our knowledge, this is the first study comparing osteoclastogenic potential of all three cell sources.

In the present study, the number of multinuclear cells on d14 was highest in PB, second highest in CB, and lowest in BM cultures. The numbers of nuclei in multinuclear cells were in line with our previous studies^19,20^. Our findings regarding the multinuclear cell number in PB and CB are in line with previous reports, which showed that CB monocytes do not fuse as efficiently as PB monocytes^14,15^. Regarding the difference in the osteoclastogenic potential of BM and CB, our results were contrary to previous report of higher number of osteoclasts in BM culture^10^. The differences can be explained by different culture conditions, as Chen et al*.* co-cultured monocyte/macrophage cells with stromal cells, 1,25-dihydroxyvitamin D_3_ and dexamethasone.

Different monocyte subsets in BM, PB, and CB can also explain the observed differences. Based on the expression of surface markers, three different monocyte subsets, classical, (CD14^++^ CD16^−^), intermediate (CD14^++^CD16^+^), and non-classical (CD14^+^ CD16^++^), have been characterized in human PB^21^. Our results were in line with previous reports on the classical monocyte being the most frequent subset in PB^22^. There are few reports on the distribution of monocytes in BM. In our study, the main subset in BM was intermediate monocytes, but classical monocytes were present almost in the same number. Classical monocytes mature in the BM and are released into circulation, where most of them live only for a short period of time, and a small portion transforms into intermediate monocytes that subsequently develop into non-classical monocytes^23^. According to some reports, the largest population of BM monocytes are CD14^++^ CD16^−^ classical monocytes, whereas others report highest proportion of intermediate CD14^++^CD16^+^ monocytes^23,24^. The pre-culture of BM samples for 1–2 days before collecting floating cells could explain differences, as it is likely that classical monocytes differentiate into intermediate and non-classical monocytes during the culture. Regarding CB, there are conflicting reports from monocyte distribution compared to adult PB. In our study, the main subset in CB was classical monocytes, but their number was lower compared to adult PB. The number of non-classical monocytes was higher in CB than PB. In other studies, the proportion of classical monocytes in CB has been reported to be higher compared to adult PB^25^. In another study, the monocyte distribution of CB was similar to adult PB^26^. In a study by Pradhu et al., the proportion of CD14^lo^ CD16^+^ patrolling monocytes was lower in CB than adult PB, but there were no differences in classical or inflammatory monocyte proportions^27^. It has been noted before that the different isolation methods can explain the conflicting results^28^. In fact, Mukherjee et al. observed that Ficoll-isolation of blood monocytes decreased the proportion of classical monocytes but increased the proportion of non-classical monosytes^29^. This phenomenon might explain our results concerning the CB monocyte subsets, but the reason why it did not affect the other cell sources remains unclear.

While monocytes of different subsets can be differentiated into osteoclasts in vitro, it has been suggested that different monocyte subsets generate functionally different osteoclasts^30,31^. In a previous study, classical osteoclasts resorbed bone more efficiently than intermediate osteoclasts, whereas non-classical osteoclasts were unable to resorb bone^32^. In the presence of pro-inflammatory cytokine IL-17A, the resorption by intermediate osteoclasts was increased whereas the resorption by classical osteoclasts remained unchanged. In the current study, BM-derived osteoclasts resorbed bone more efficiently than PB and CB osteoclasts and the proportion of intermediate monocytes was greater in BM. Our hypothesis is that osteoclasts derived from intermediate monocytes react in a similar way in vitro as in a pro-inflammatory setting.

Foreign body giant cells are multinucleated cells originating from monocyte/macrophage lineage cells that fuse when they interact with foreign bodies^33^. TRACP-positive foreign body giant cells share characteristics with osteoclast, but foreign body cells have a higher nuclei number and they do not resorb bone^34,35^. In the current study, the multinuclear cells in the PB and CB cultures had higher number of nuclei compared to BM cultures. In addition, BM cultures had higher resorption activity than PB and CB cultures. These differences could mean that some of the multinuclear cells in PB and CB cultures are giant cell macrophages instead of osteoclasts. In PB and CB cultures, the presence of bone slices might cause the foreign body reaction since the in vitro culture with devitalized bone does not fully represent the in vivo bone tissue. The explanation why foreign body reaction did not occur in BM cultures could be the different monocyte subsets in the cell sources.

The starting point of the monocyte fusion is difficult to predict, but some estimates have been suggested in literature. Fusion of PB monocytes has been reported to begin approximately on culture day 3–4, while BM monocytes have been reported to start fusion on days 5–10^36–41^. Our own experience is in line with these studies, and therefore, we chose d8 as the first time point for counting multinuclear cell numbers. However, there is limited data on the course and completion of the fusion. Our current research shows that the increase in the number of BM-derived monocytes is highest between d8–d11 and of PB-derived between d11–d14. This finding could indicate that osteoclastogenesis is completed earlier in the BM-derived cultures, which is in line with our previous study comparing PB and BM, and a study comparing BM and CB^5,19^. In addition, the higher number of nuclei in multinuclear cells in PB and CB indicates that osteoclastogenesis continues for a longer time in blood- than BM-derived cells. Experiments of long-term osteoclast cultures exceeding the length of standard osteoclast culture protocols could explain our observations. For instance, in a^42^ RAW 264.7 cell culture continued for up to 26 days, an oscillatory pattern in osteoclast fusion and apoptosis was found^42^. In similar conditions to our study, it was observed that in the PB monocyte cultures, the number of osteoclasts is highest on d14 and declines by d21^43^. When human PB monocytes were cultured for 21 days, it was observed that the onset of osteoclast resorption occurred between day 4 and day 7 and further increased up to day 16 and then decreased until the end of the experiment at 21 days^44^.

As we have previously reported, a significant proportion of cells in BM cultures were fibroblast-shaped stromal-like cells^19^. As these spindle-like cells are TRACP-negative and morphologically different from the spherical monocytes, we assume that they represent the mesenchymal stromal cell (MSC) population. There are also mesenchymal cells in PB and CB, but their number is lower^45,46^. The presence of MSCs is likely to explain some of the observed differences between BM and blood-derived cultures.

In bone, osteoblasts and osteocytes regulate osteoclastogenesis by complex mechanisms. Osteocytes and osteoblasts produce proteins that are essential for osteoclastogenesis, such as M-CSF, which promotes pre-osteoclast proliferation and differentiation; RANKL, which acts mainly as a surface protein but occurs also in a soluble form and activates fusion and differentiation of osteoclasts; and OPG, which inhibits osteoclastogenesis and the subsequent bone remodeling^47–49^. Moreover, RANKL signaling is probably bidirectional: RANKL also increases osteoblast differentiation^50^. RANKL/OPG ratio is important in bone homeostasis^51^. In general, the cytokine concentrations in the present study showed overall fluctuating pattern over time, which probably reflects the fluctuating activation of osteoclasts and mesenchymal stromal cells. Increasing RANKL concentration from 20 to 80 ng/ml caused only a slight increase in the number of multinuclear cells and resorption pits. In RANKL 80 ng/ml cultures the proportion of soluble RANKL compared to membrane-bound is increased, because it is added in the media. In the RANKL 20 ng/ml group, it is possible, that the stromal cells produce more membrane-bound form of RANKL. In a previous study, membrane-bound RANKL worked more efficiently than soluble^52^. RANKL stimulates osteoclastogenesis in a dose-dependent manner, but the effect is saturated with relatively low RANKL concentrations. Increasing RANKL concentration from 10 to 100 ng/ml did not seem to enhance osteoclastogenesis in previous studies^48,53^. In the present study, the OPG concentrations during the culture differed between RANKL 20 ng/ml and 80 ng/ml groups. The effect was most notable in BM cultures. This could be caused by the high RANKL 80 ng/ml concentration exceeding the optimal physiological level that changes the cytokine equilibrium. The concentration of M-CSF was highest in BM RANKL 20 ng/ml cultures at time points d8 and d11. One explanation for this could be that stromal cells produce M-CSF to enhance osteoclastogenesis.

In the present study, OPG concentrations in the cultures without growth factors fluctuated similarly in all cell sources. The peaking of OPG in BM RANKL 20 ng/ml group could be caused by physiological inhibition of osteoclastogenesis by stromal cells when the number of osteoclasts is high and the resorption is active, as the conditions in this culture probably bear the most resemblance to the physiological situation. In addition to stromal cells, OPG is expressed in B lymphocytes, dendritic cells, and follicular dendritic cells^54^. In our study, B lymphocytes can exist in collected samples from all cell sources and dendritic cells can be differentiated from precursors in all cultures. Similar OPG concentrations and bone resorption were not observed in PB and CB cultures, which suggest that the observation is related to the presence of stromal cells.

Some growth factors can have different properties depending on the density and differentiation stage of the osteoclasts. Co-culture with MSCs can enhance or inhibit the differentiation of osteoclasts depending on the inflammatory state of the culture via soluble factors and cell-to-cell interactions^55^. The higher M-CSF concentration in CB culture on d4 could be explained by the presence of stromal cells that do not survive very long in standard cell culture conditions but might be active in the beginning of the culture by secreting M-CSF^46,56^.

In the present study, infrequent spontaneous osteoclastogenesis occurred in all cultures. The number of nuclei in spontaneously fused osteoclast was only slightly lower compared to cultures with growth factors. This could suggest that in the absence of RANKL and M-CSF, the fusion of osteoclasts may start but stops at a certain point if the conditions are disadvantageous.

This study could not replicate the previous finding of spontaneous osteoclastogenesis of umbilical cord monocytes. One possible explanation could be the presence of stromal cells in previous studies^10,17^. It is likely that our method does not enable sufficient survival of the stromal cells in the CB culture. One possible explanation is also the difference between monocyte donor selection, as we saw great variation in the osteoclastogenic potential between the cultures.

Due to practical reasons and coincidence all our CB monocyte cultures were from female newborn donors. Contradictory results have been published concerning the osteoclastogenic potential of male and female monocytes^57^. A recent study suggests that there is wide variation between OCs differentiated from different donors’ PBMCs^58^. We have also noticed this in the present and our previous studies, and therefore, have included BM and PB samples from both sexes to every experiment in the study. The collection of human samples from patients undergoing surgery cannot be planned in detail concerning the age and sex of the patients, but we have tried to overcome this issue by collecting as many samples as possible.

## Conclusion

In conclusion, CB-derived osteoclasts showed characteristics that were in between BM and PB-derived osteoclasts, though they resembled more PB-derived cells. The presence of stromal cells in CB could cause the observed shift from PB towards BM, as we expect that the stromal cells in CB act similarly to stromal cells present in BM, where they participate in osteoclastogenesis. It is too early to draw any conclusions of the clinical or in vivo significance of the reported phenomenon.

The collection of BM and PB samples from human patients is invasive and requires laborious scheduling. The advantage of using CB samples for cultures is that it is obtained from a residual material and might be more easily available, although there are issues that cannot be resolved when obtaining CB samples, such as the unpredictable sex of the newborn. We show that functional osteoclasts can be differentiated from BM, PB, and CB monocytes, even though there are differences in their properties and function.

## Data Availability

The data that was analyzed during the current study are available on request from the corresponding author.

## References

[1] Matsuzaki K (1998). Osteoclast differentiation factor (ODF) induces osteoclast-like cell formation in human peripheral blood mononuclear cell cultures. Biochem. Biophys. Res. Commun..

[2] Udagawa N (1990). Origin of osteoclasts: Mature monocytes and macrophages are capable of differentiating into osteoclasts under a suitable microenvironment prepared by bone marrow-derived stromal cells. Proc. Natl. Acad. Sci. U.S.A..

[3] Xing L, Schwarz EM, Boyce BF (2005). Osteoclast precursors, RANKL/RANK, and immunology. Immunol. Rev..

[4] Shalhoub V (2000). Characterization of osteoclast precursors in human blood. Br. J. Haematol..

[5] Eeles DG (2015). Osteoclast formation elicited by interleukin-33 stimulation is dependent upon the type of osteoclast progenitor. Mol. Cell. Endocrinol..

[6] Hodge JM (2004). Osteoclastic potential of human CFU-GM: Biphasic effect of GM-CSF. J. Bone Miner. Res..

[7] Kalantari N (2016). Effect of the receptor activator of nuclear factor кB and RANK ligand on in vitro differentiation of cord blood CD133(+) hematopoietic stem cells to osteoclasts. Cell J..

[8] Sawano A (2001). Flt-1, vascular endothelial growth factor receptor 1, is a novel cell surface marker for the lineage of monocyte-macrophages in humans. Blood.

[9] Roux S (1997). Effects of prostaglandins on human hematopoietic osteoclast precursors. Endocrinology.

[10] Chen FP, Wang KC, Huang JD (2009). Effect of estrogen on the activity and growth of human osteoclasts in vitro. Taiwan. J. Obstet. Gynecol..

[11] Park HC (2017). Effects of osteogenic-conditioned medium from human periosteum-derived cells on osteoclast differentiation. Int. J. Med. Sci..

[12] Penolazzi L (2008). Human osteoclasts differentiated from umbilical cord blood precursors are less prone to apoptotic stimuli than osteoclasts from peripheral blood. Apoptosis.

[13] Quinn JM, Fujikawa Y, McGee JO, Athanasou NA (1997). Rodent osteoblast-like cells support osteoclastic differentiation of human cord blood monocytes in the presence of M-CSF and 1,25 dihydroxyvitamin D3. Int. J. Biochem. Cell Biol..

[14] Gerberding K, Yoder MC (1993). In vitro comparison of multinucleated giant cell formation from human umbilical cord and adult peripheral blood mononuclear phagocytes. Pediatr. Res..

[15] Kondo Y (2009). Multi-nucleated giant cell formation from human cord blood monocytes in vitro, in comparison with adult peripheral blood monocytes. Clin. Exp. Immunol..

[16] Erices A, Conget P, Minguell JJ (2000). Mesenchymal progenitor cells in human umbilical cord blood. Br. J. Haematol..

[17] Sun B (2007). Regulation of human umbilical cord blood-derived multi-potent stem cells by autogenic osteoclast-based niche-like structure. Biochem. Biophys. Res. Commun..

[18] Mira Susa, Ngoc-Hong Luong-Nguyen, David Cappellen, Natasa Zamurovic & Rainer Gamse *et al.* Human primary osteoclasts: in vitro generation and applications as pharmacological and clinical assay. *J. Transl. Med.* **2**, 6 (2004). 10.1186/1479-5876-2-6 PMC394349 15025786

[19] Kylmäoja E (2018). Peripheral blood monocytes show increased osteoclast differentiation potential compared to bone marrow monocytes. Heliyon.

[20] Kylmäoja E (2018). Gap junctional communication is involved in differentiation of osteoclasts from bone marrow and peripheral blood monocytes. Heliyon.

[21] Ziegler-Heitbrock L (2010). Nomenclature of monocytes and dendritic cells in blood. Blood.

[22] Wong KL (2011). Gene expression profiling reveals the defining features of the classical, intermediate, and nonclassical human monocyte subsets. Blood.

[23] Patel AA (2017). The fate and lifespan of human monocyte subsets in steady state and systemic inflammation. J. Exp. Med..

[24] Mandl M, Schmitz S, Weber C, Hristov M (2014). Characterization of the CD14^++^CD16^+^ monocyte population in human bone marrow. PLoS One.

[25] Damasceno D (2019). Distribution of subsets of blood monocytic cells throughout life. J. Allergy Clin. Immunol..

[26] Sohlberg E, Saghafian-Hedengren S, Bremme K, Sverremark-Ekström E (2011). Cord blood monocyte subsets are similar to adult and show potent peptidoglycan-stimulated cytokine responses. Immunology.

[27] Prabhu SB (2016). Comparison of human neonatal and adult blood leukocyte subset composition phenotypes. PLoS One.

[28] Wong KL (2012). The three human monocyte subsets: Implications for health and disease. Immunol. Res..

[29] Mukherjee R (2015). Non-classical monocytes display inflammatory features: Validation in sepsis and systemic lupus erythematous. Sci. Rep..

[30] Komano Y, Nanki T, Hayashida K, Taniguchi K, Nobuyuki M (2006). Identification of a human peripheral blood monocyte subset that differentiates into osteoclasts. Arthritis Res. Ther..

[31] Das A (2021). Monocyte subsets with high osteoclastogenic potential and their epigenetic regulation orchestrated by IRF8. J. Bone Miner. Res..

[32] Sprangers S, Schoenmaker T, Cao Y, Everts V, de Vries TJ (2016). Different blood-borne human osteoclast precursors respond in distinct ways to IL-17A. J. Cell. Physiol..

[33] Anderson JM, Rodriguez A, Chang DT (2008). Foreign body reaction to biomaterials. Semin. Immunol..

[34] ten Harkel B (2015). The foreign body giant cell cannot resorb bone, but dissolves hydroxyapatite like osteoclasts. PLoS One.

[35] Khan UA, Hashimi SM, Bakr MM, Forwood MR, Morrison NA (2013). Foreign body giant cells and osteoclasts are TRAP positive, have podosome-belts and both require OC-STAMP for cell fusion. J. Cell. Biochem..

[36] Massey HM, Flanagan AM (1999). Human osteoclasts derive from CD14-positive monocytes. Br. J. Haematol..

[37] Lader CS, Scopes J, Horton MA, Flanagan AM (2001). Generation of human osteoclasts in stromal cell-free and stromal cell-rich cultures: Differences in osteoclast CD11c/CD18 integrin expression. Br. J. Haematol..

[38] Mira-Pascual L (2020). A novel sandwich ELISA for tartrate-resistant acid phosphatase 5a and 5b protein reveals that both isoforms are secreted by differentiating osteoclasts and correlate to the type I collagen degradation marker CTX-I in vivo and in vitro. Calcif. Tissue Int..

[39] Møller AMJ, Delaissé JM, Søe K (2017). Osteoclast fusion: Time-lapse reveals involvement of CD47 and syncytin-1 at different stages of nuclearity. J. Cell. Physiol..

[40] Flanagan AM, Massey HM (2003). Generating human osteoclasts in vitro from bone marrow and peripheral blood. Methods Mol. Med..

[41] Cody JJ (2011). A simplified method for the generation of human osteoclasts in vitro. Int. J. Biochem. Mol. Biol..

[42] Akchurin T (2008). Complex dynamics of osteoclast formation and death in long-term cultures. PLoS One.

[43] Abdallah D (2018). An optimized method to generate human active osteoclasts from peripheral blood monocytes. Front. Immunol..

[44] de MeloPereira D, Davison N, Habibović P (2022). Human osteoclast formation and resorptive function on biomineralized collagen. Bioact. Mater..

[45] He Q, Wan C, Li G (2007). Concise Review: Multipotent mesenchymal stromal cells in blood. Stem Cells.

[46] Lee OK (2004). Isolation of multipotent mesenchymal stem cells from umbilical cord blood. Blood.

[47] Kodama H, Nose M, Shumpei N, Yamasaki A (1991). Essential role of macrophage colony-stimulating factor in the osteoclast differentiation supported by stromal cells. J. Exp. Med..

[48] Lacey DL (1998). Osteoprotegerin ligand is a cytokine that regulates osteoclast differentiation and activation. Cell.

[49] Simonet WS, Lacey DL, Dunstan CR (1997). Osteoprotegerin: A novel secreted protein involved in the regulation of bone density. Cell.

[50] Cao X (2018). RANKL-RANK signaling regulates osteoblast differentiation and bone formation. Bone Res..

[51] Boyce BF, Xing L (2007). Biology of RANK, RANKL, and osteoprotegerin. Arthritis Res. Ther..

[52] Nakashima T (2000). Protein expression and functional difference of membrane-bound and soluble receptor activator of NF-κB ligand: Modulation of the expression by osteotropic factors and cytokines. Biochem. Biophys. Res. Commun..

[53] Li J (2000). RANK is the intrinsic hematopoietic cell surface receptor that controls osteoclastogenesis and regulation of bone mass and calcium metabolism. PNAS.

[54] Yun TJ (1998). OPG/FDCR-1, a TNF receptor family member, is expressed in lymphoid cells and is up-regulated by ligating CD40. J. Immunol..

[55] Sharaf-Eldin WE, Abu-Shahba N, Mahmoud M, El-Badri N (2016). The modulatory effects of mesenchymal stem cells on osteoclastogenesis. Stem Cells Int..

[56] Laitinen A, Nystedt J, Laitinen S (2011). The isolation and culture of human cord blood-derived mesenchymal stem cells under low oxygen conditions. Methods Mol. Biol..

[57] Lorenzo J (2020). Sexual dimorphism in osteoclasts. Cells.

[58] Møller AMJ (2020). Zoledronic acid is not equally potent on osteoclasts generated from different individuals. JBMR Plus.

